# Effects of extrathoracic mechanical ventilation on pulmonary hypertension secondary to lung disease

**DOI:** 10.1007/s00540-016-2172-7

**Published:** 2016-04-18

**Authors:** Yoko Sato, Noriyuki Saeki, Takuma Asakura, Kazutetsu Aoshiba, Toru Kotani

**Affiliations:** Department of Anesthesiology and Intensive Care Medicine, Tokyo Women’s Medical University, 8-1 Kawada-cho, Shinjuku-ku, Tokyo, 162-8666 Japan; Synthesis Shinkawabashi Hospital, 1-15 Shinkawadori, Kawasaki-Ku, Kawasaki, Kanagawa 210-0013 Japan; Nitta Central Clinic, 1-20-19 Yaguchi, Ohta-ku, Tokyo, 146-0093 Japan; Department of Respiratory Medicine, Tokyo Medical University Ibaraki Medical Center, 3-20-1 Chuo, Ami, Inashiki, Ibaraki 300-0395 Japan

**Keywords:** Biphasic cuirass ventilation, Pulmonary hypertension, Ventilator

## Abstract

**Purpose:**

Biphasic cuirass ventilation (BCV) is a form of non-invasive extrathoracic positive and negative pressure mechanical ventilation. The present study was conducted to quantify our positive experience using BCV to dramatically improve gas exchange and cardiac function in patients with acute exacerbation of chronic respiratory failure and secondary pulmonary hypertension (PH).

**Methods:**

BCV was applied for 2 weeks in 17 patients with PH caused by lung disease. Ventilation sessions were limited to 1 h per day to prevent exhaustion. To assess respiratory and circulatory effects, percutaneous arterial oxygen saturation (SpO_2_) was measured before and after each daily BCV session, and right heart catheter test [mean pulmonary artery pressure (mPAP), right atrium pressure (RAP), pulmonary artery occlusion pressure (PAOP) and cardiac index (CI)] and serum N-terminal pro-brain natriuretic peptide (NT-proBNP) were measured before and after a series of BCV sessions.

**Results:**

SpO_2_ transiently improved after each BCV session. After a series of BCV, mPAP decreased from 27.2 to 22.4 mmHg (*p* = 0.0007). PAOP, CI and serum NT-proBNP levels decreased compared with baseline. No patients were treated with epoprostenol, iloprost, bosentan or sildenafil for PH.

**Conclusion:**

BCV may improve circulatory function in patients with PH caused by lung disease.

**Electronic supplementary material:**

The online version of this article (doi:10.1007/s00540-016-2172-7) contains supplementary material, which is available to authorized users.

## Introduction

Structural and functional alterations of pulmonary vessels eventually lead to secondary pulmonary hypertension (PH) and cor pulmonale [1] in patients with chronic lung disease. These alterations are considered to be the end stage of chronic lung disease and the only effective treatment currently available involves supplemental oxygen, diuretics, digitalis and anticoagulants [1–3]. Recently, prostanoids, endothelin receptor antagonists and phosphodiesterase-5 inhibitors have been tested as active treatments; however, their effectiveness remains controversial [4–7]. Guidelines published by the European Society of Cardiology/European Respiratory Society do not recommend secondary PH as a target for treatment if pulmonary artery pressure (PAP) is <40 mmHg, citing a lack of available data regarding treatment safety and efficacy [8]. Long-term oxygen therapy has been effective in improving the physical condition of patients with chronic obstructive pulmonary disease (COPD) and PAP <40 mmHg; however, use of long-term oxygen therapy does not contribute to prevention of COPD progression, including secondary PH [9].

Biphasic cuirass ventilation (BCV), a form of non-invasive extrathoracic mechanical ventilation, is an advanced model of the iron lung. It consists of a cuirass and a pressure source capable of providing both positive and negative pressure, which supports spontaneous breathing in a more physiological manner compared with positive pressure ventilation. BCV remains in clinical use and has been shown to be as effective as non-invasive positive pressure ventilation for avoiding tracheal intubation in COPD patients [10].

Negative pressure ventilation (NPV) provided by BCV increases pulmonary blood flow after the Fontan operation [11] and improves oxygenation in newborn infants with persistent PH [12]. We have experienced dramatic improvements in gas exchange and cardiac function in adult patients with acute exacerbation of chronic respiratory failure and secondary PH when applying BCV [13]. Based on our experience and observations, we hypothesized that BCV provides better oxygenation than baseline and reduces pulmonary vascular resistance (PVR) in PH caused by chronic lung disease. In the present study, we examined this hypothesis in patients undergoing BCV as a treatment for PH secondary to lung disease.

## Methods

This observational study involved patients recruited from Tokyo Women’s Medical University Hospital and Synthesis Shinkawabashi Hospital in Japan. The study was conducted from April 2009 until April 2012 using a protocol approved by the ethics committee from each hospital. Written informed consent was obtained from all patients.

PH was defined as mean pulmonary artery pressure (mPAP) >20 mmHg in accordance with guidelines from the Japanese Cardiac Society (2006). Steady-state PAP was measured by right heart catheterization. Exclusion criteria were (1) PH caused by idiopathic pulmonary arterial hypertension, connective tissue disease, chronic thromboembolism, portal hypertension, congenital heart disease, left heart disease, chronic hemolytic anemia, HIV infection or other unknown reason; (2) acute exacerbation of chronic lung disease; (3) unstable hemodynamics (such as acute heart failure, arrhythmia or myocardial ischemia); (4) infection of any organ; (5) pulmonary vasodilator medication, such as epoprostenol, iloprost, bosentan, or sildenafil; (6) failure in cuirass attachment due to a skin problem; and (7) otherwise judged by a physician as inappropriate for study inclusion.

### Patient selection

Patients who regularly visited the hospital with dyspnea secondary to chronic lung disease were diagnosed with PH using ultrasonic echocardiography. First, right heart catheterization was performed and PH was definitively diagnosed during an approximate 2-week admission. Hemodynamics were measured at the same time (prior to starting a series of BCV sessions). BCV was performed on consecutive weekdays and a second right-heart catheterization was performed just before hospital discharge to compare hemodynamics before and after BCV. Patient chest X-ray, blood analysis and pulmonary function tests were rechecked for comparison with results from the day of admission.

### Right heart catheterization

Right heart catheterization was performed at the beginning of an approximate 2-week admission. In each test, electrocardiograph and percutaneous arterial oxygen saturation (SpO_2_) were continuously monitored. Noninvasive blood pressure was recorded every 5 min and arterial blood gas was analyzed. Central venous pressure, right atrial pressure (RAP), PAP, pulmonary artery occlusion pressure (PAOP) and cardiac index (CI) were measured using a thermodilution catheter with jugular venous access. mPAP and PVR were calculated using the formula: mPAP = diastolic PAP + (systolic PAP − diastolic PAP)/3, PVR = (mPAP − PAOP)/cardiac output.

### BCV session

BCV was performed every weekday during admission using an RTX ventilator (United Hayek Medical, London, UK). To promote biphasic pressure with a time cycle, the control mode was used as an initial setting with a negative pressure from −15 to −25 cmH_2_O (synchronized to each patient’s inspiration) and a positive pressure from 3−10 cmH_2_O (synchronized for expiration). BCV sessions were limited to 30–60 min/day to prevent exhaustion. Ventilatory rate and inspiratory:expiratory ratio were set and adjusted to synchronize with spontaneous breathing. To maintain stable conditions and record changes in oxygenation, SpO_2_ was continuously monitored for several minutes prior to starting and after completing BCV.

### Blood samples

Venous blood samples were taken at admission and before discharge. Complete blood count, serum concentrations of N-terminal proB-type natriuretic peptide (NT-proBNP) and noradrenalin were measured for comparison.

### Treatment of PH

Pharmacological treatments initiated prior to admission were not interrupted during the course of study. Long-term oxygen therapy was maintained on one patient, with the flow dose of oxygen remaining unchanged. After the BCV study, all patients were asked the following three questions—Was the BCV comfortable? Did the BCV improve your dyspnea? Would you like to use the BCV at an outpatient clinic?

### Statistical analysis

Statistical analysis was performed using JMP software, version 11.2.0 (SAS Institute, Cary, NC, USA). Normally distributed numerical data are expressed as mean and standard deviation (SD) values, whereas skewed data are expressed as median and interquartile range. Qualitative data are expressed as number and percentage. For normally distributed numerical data, Student’s *t* test was employed; whereas, for skewed numerical data, a Wilcoxon rank sum test was applied. SpO_2_ changes were analyzed using Kruskal–Wallis test. Two-sided *P* values <0.05 were considered statistically significant.

## Results

Seventeen patients with steady-state PH caused by chronic lung disease were included in the study; Table 1 illustrates cohort characteristics. Table 2 indicates relevant characteristics of the two sub-classified patient groups. The restriction group included post-thoracoplasty for treatment of tuberculosis (*n* = 3), interstitial pneumonia (*n* = 1) or pulmonary asbestosis (*n* = 1). The COPD group included individuals at various stages of Global Initiative for Chronic Obstructive Lung Disease (GOLD; GOLD 2, *n* = 2; GOLD 3, *n* = 2; and GOLD 4, *n* = 7).

**Table 1:** Patient characteristics

Age, mean (±SD) years	70.1 ± 8.7
Male, *n* (%)	15 (88)
BMI, mean (±SD) kg/m^2^	19.1 ± 3.5
Disease, *n* (%), COPD	11 (64.7)
Post thoracoplasty	3 (17.6)
Others (old tuberculosis, asbestosis, interstitial pneumonia)	3 (17.6)

*BMI* body mass index, *COPD* chronic obstructive pulmonary disease

**Table 2:** Patient characteristics of the two groups

	COPD	Restriction
*n*	11	5
Age (years)	68.6 ± 9	69.4 ± 5.4
BMI	18.2 ± 3.8	20.2 ± 2.1
Weight (kg)	48.8 ± 12.8	51.4 ± 5.2
%VC	80.8 (64.8–97.6)	52.1 (47.7–58.5)
FEV1.0 (L)	0.78 (0.67–0.99)	1.09 (0.81–1.33)

*%VC* vital capacity, *FEV1.0* forced expiratory volume in one second

Of the 17 patients, 15 underwent catheterization twice and a series of BCV sessions during an approximate 2-week admission. The remaining two patients underwent right heart catheterization first, but were discharged before BCV because of financial reasons or the Great East Japan Earthquake; however, they were readmitted to the hospital for BCV. The number and final timing of BCV sessions administered during an approximate 2-week admission (Table 3a, b) varied because of the availability of BCV equipment and catheter laboratory resources.

**Table 3:** (a) Number of BCV sessions, (b) Final timing of BCV session of the second catheterization

	COPD	Restriction
(a) Number of BCV sessions		
8 times	2	1
9	1	1
10	4	3
11	3	0
12	1	0
(b) Final timing of BCV session of the second catheterization		
1 day before	6	2
On the morning	5	3

**Fig. 1:**
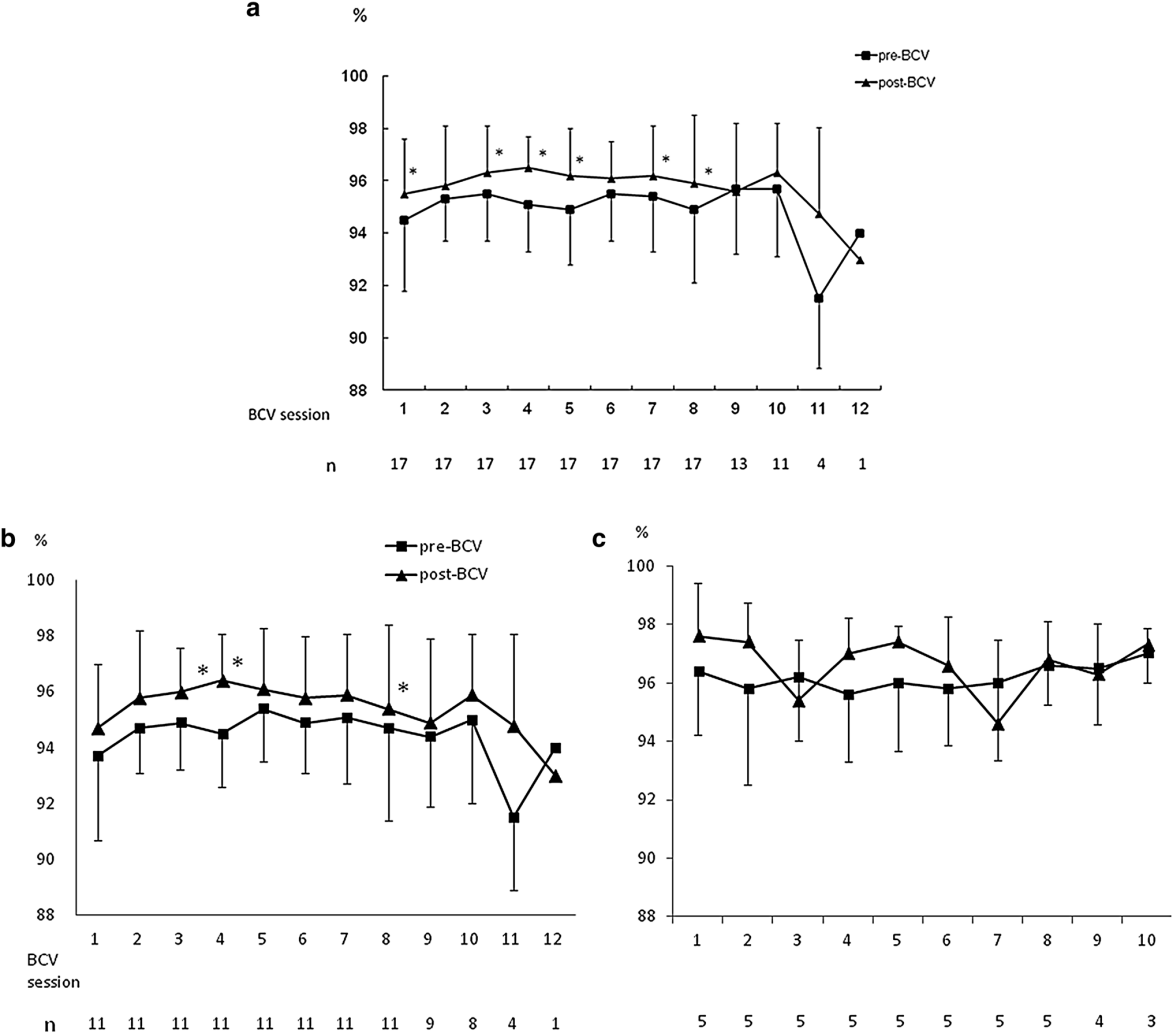
Saturation of peripheral oxygen (SpO_2_) before and after daily biphasic cuirass ventilation (BCV) sessions. **a** SpO_2_ of total cases. **b** SpO_2_ of chronic obstructive pulmonary disease (COPD) group. **c** SpO_2_ of restriction group. *Closed box* pre-BCV; *closed triangle* post-BCV; *n* patient number. BCV transiently improved oxygenation in total cases and COPD group. *P < 0.05 versus pre-BCV

**Fig. 2:**
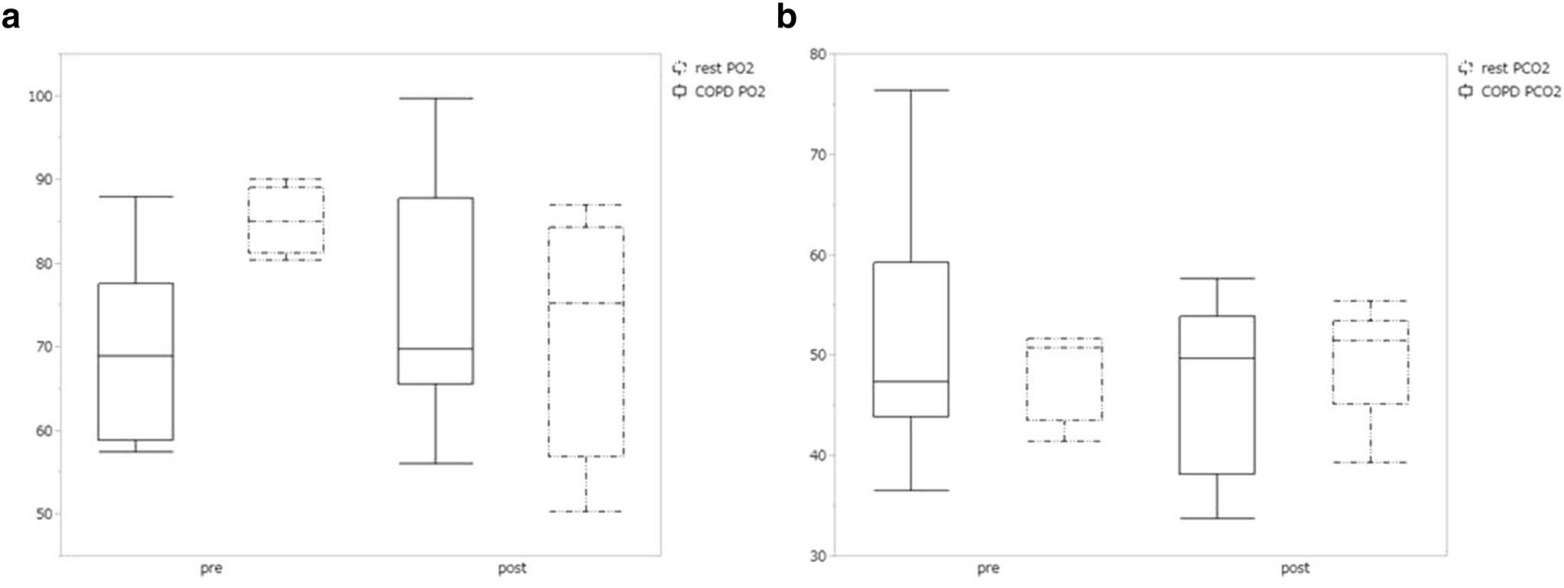
Changes in partial pressure of oxygen (PaO_2_) (**a**) and partial pressure of carbon dioxide (PaCO_2_) (**b**) before and after a series of BCV sessions. Arterial blood gas was measured at the same time as right heart catheterization, and no statistically significant differences were found. *Box* represents interquartile range and *horizontal line across the box* represents median. *Error bars* represent minimum and maximum. COPD group, *simple line box*; restriction group, *dot line box*

BCV improved oxygenation after each session; however, this effect was transient and SpO_2_ returned to baseline levels by the following day (Fig. 1a–c). Furthermore, after a series of BCV sessions, PaO_2_ and PaCO_2_ remained unchanged compared with baseline levels (Fig. 2). BCV significantly decreased mPAP (27.2 ± 5.1 to 22.4 ± 4.9 mmHg, *P* = 0.0007), RAP (8.5 ± 4.8 to 4.7 ± 3.9 mmHg, *P* = 0.0044), PAOP (13.7 ± 5.6 to 8.8 ± 4.7 mmHg, *P* = 0.0041) and CI (2.7 ± 0.6 to 2.4 ± 0.5 l/min/m^2^, *P* = 0.0309), whereas PVR was unchanged (267.6 ± 114.5 to 288.0 ± 100.5 dyne/s/m^5^, *P* = 0.67).

**Fig. 3:**
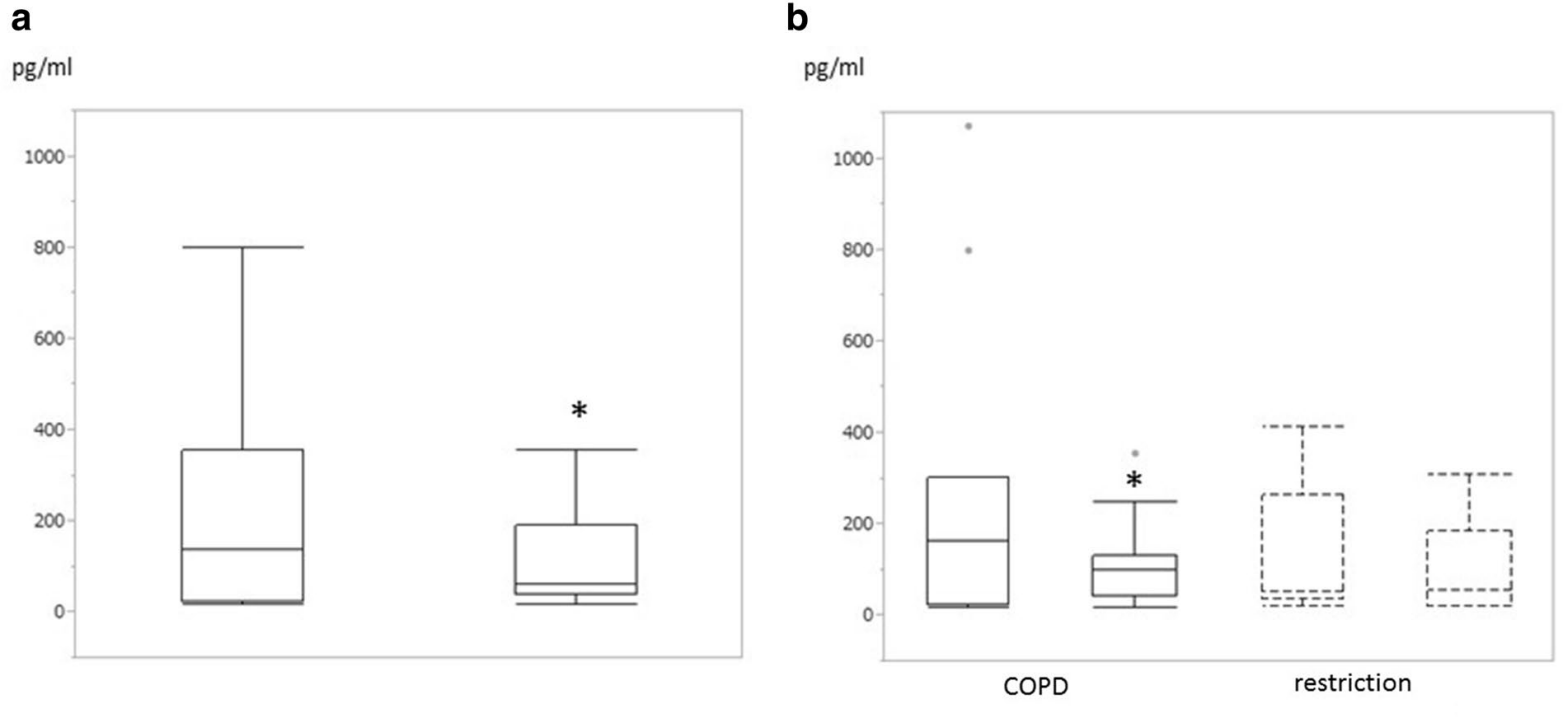
Changes in serum N-terminal proB-type natriuretic peptide (NT-proBNP) before and after a series of BCV sessions. **a** NT-proBNP decreased significantly after BCV in all cases (*P* < 0.05). **b** NT-proBNP significantly decreased in the COPD group but not in the restriction group. COPD group, *simple line box*; restriction group, *dot line box*. *Box* represents interquartile range and *horizontal line across the box* represents median. *Error bars* represent minimum and maximum. *Dots* represent outlier. *P < 0.05

A significant decrease was observed in serum NT-proBNP levels [136.5 (23.5–357.4) to 60.2 (39.4–190) pg/ml, *P* = 0.0056, Fig. 3a], but not noradrenalin levels [411 (291–784) to 389.5 (295–765.8) pg/ml, *P* = 0.599]. Mean blood pressure decreased significantly after each BCV session (Table 5).

In subgroup analysis between COPD and restriction groups, mPAP decreased in both (COPD, *P* = 0.0167; restriction, *P* = 0.0481; Table 4). However, RAP (*P* = 0.003), PAOP (*P* = 0.007; Table 4) and serum NT-proBNP levels decreased only in the COPD group [162 (22.6–302.3) to 100.8 (43.9–131) pg/ml, *P* = 0.042; Fig. 3b]. CI and PVR did not change (Table 4); however, subgroup analysis indicated blood pressure, heart rate and noradrenalin remained unchanged (Table 5).

**Table 4:** Changes in parameters of right heart catheterization before and after 2-week BCV sessions in subgroup analysis

	mPAP	RAP	PAOP	CI	PVR
COPD					
Pre	26.5 ± 5.0	9.3 ± 4.0	13.3 ± 5.1	2.6 ± 0.5	264.6 ± 93.0
Post	22.8 ± 5.3*	4.6 ± 3.9*	8.6 ± 3.0*	2.4 ± 0.4	302.9 ± 77.3
Restriction					
Pre	28.4 ± 6.2	5.0 ± 4.3	13.8 ± 7.5	3.0 ± 0.6	275.9 ± 175.2
Post	21.2 ± 4.7*	5.0 ± 4.7	6.2 ± 2.6	2.5 ± 0.6	308.0 ± 83.8

*mPAP* mean pulmonary arterial pressure, *RAP* right atrial pressure, *PAOP* pulmonary artery occlusion pressure, *CI* cardiac index, *PVR* pulmonary vascular resistance

* *P* < 0.05

**Table 5:** Changes in blood pressure and heart rate

	Pre BCV	Post BCV	*P* value
Mean BP (mmHg) all cases	90.5 ± 8.7	86.0 ± 7.1	0.0061
COPD	78.1 ± 16.8	84.9 ± 7.5	0.0698
Restriction	92.2 ± 9.1	87.8 ± 7.4	0.1079
Heart rate (beats/min) all cases	83.2 ± 17.9	81.1 ± 14.7	0.507
COPD	78.1 ± 16.8	80.3 ± 12.3	0.4896
Restriction	97.2 ± 14.7	86.2 ± 19.6	0.199

After the BCV study, all restriction group patients requested to take the device home because they felt comfortable and their dyspnea was almost completely attenuated. Ten other patients indicated that their dyspnea improved but the trial was uncomfortable. Two COPD patients wanted to cease BCV because of discomfort, but cooperated for study completion.

## Discussion

BCV was applied for 2 weeks in 17 patients with PH caused by lung disease. SpO_2_ after each daily BCV session improved transiently, but it was not enough to improve gas exchange even after a series of BCV sessions. After a series of BCV sessions, mPAP, RAP, PAOP and CI measured by right heart catheterization and serum NT-proBNP levels decreased compared with the baselines, whereas PVR did not. In subgroup analysis between COPD and restriction groups, only mPAP decreased in both groups.

It is obvious that hypoxemia and hypercapnia are major causes of PH; thus, long-term oxygen therapy or non-invasive positive pressure ventilation has been used for patients with chronic lung diseases. A previous study reported long term non-invasive positive pressure ventilation improved gas exchange both in COPD and restriction groups, whereas mPAP only decreased within the restriction group [14]. Transient improvement of SpO_2_ by BCV possibly benefited patients in our study; however, these patients were stable and did not present with acute exacerbation of lung disease. Furthermore, BCV decreased mPAP in both the COPD and restriction group, suggesting it is unlikely that correction of hypoxemia and hypercapnia by BCV was the only cause of reduced mPAP.

There are two possible mechanisms by which BCV ameliorates PH. First, BCV may directly improve thoracic mobility. In the present study, ventilation measurements during BCV were not performed. However, many studies have shown tidal volume and minute volume increase with the use of NPV or BCV in healthy volunteers [15, 16], as well as in patients with chest wall diseases [17] and COPD [18]. Previous studies demonstrate that negative pressure relieves the diaphragm and other respiratory muscles in COPD patients [18, 19].

Extrathoracic negative pressure directly affects expansion of the thoracic cage by stretching the diaphragm and intercostal muscles for inspiration (see Online Resource 1). Improvement of thoracic mobility increases ventilation volumes and allows for comfortable respiration in restrictive lung disease patients. Indeed, all restriction group patients requested further BCV therapy after discharge. Extra-thoracic positive pressure during expiration assisted return to resting position and may assist in elastic recoil of the chest wall (see Online Resource 1).

Pressure swings caused by airflow limitation and reduction of the vascular bed have been shown to elevate PAP in COPD patients [1, 20]. Severe airflow limitation in COPD patients with normal left ventricular function results in superimposed pressure in both the left and right ventricles caused by auto-positive end expiratory pressure. An increase in both intrathoracic pressure [1] and dynamic hyperinflation [21] has been shown to increase PAOP, which acts interdependently with RAP [22]. Grasso and colleagues reported that NPV provides more effective lung inflation volume uniformity during both inspiration and expiration compared with positive pressure ventilation in an acute lung injury model in rabbits [23]. NPV, when used for high-frequency ventilation, has been shown to lower intrathoracic negative pressure [24, 25]. Thus, if BCV decreases intrathoracic pressure, eventually extinguishes auto-positive end expiratory pressure and also provides uniform ventilation, it decreases PAOP as well as RAP. Because the stroke index parallels PAOP, BCV mitigates the workload of both ventricles, leading to a decrease of whole parameters in the right cardiopulmonary unit.

Second, BCV may decrease the work required for breathing by influencing interactions between respiration and circulation. CI decreased statistically, but data recovered to normal ranges. BCV may decrease cardiac load to reduce excess work of breathing associated with chronic lung disease and decreased serum NT-proBNP. It is highly likely that mitigation of breathing workload normalizes CI. Furthermore, improvement in thoracic mobility by BCV, both inspiratory and expiratory support, correlates with decreased work of breathing.

We anticipated PVR would decrease during this study, but results from our series of BCV sessions indicated unchanged PVR in all cases and subgroup analyses. Whole parameters, including CI, significantly decreased in the right cardiopulmonary unit, i.e., data recovered to within normal ranges. In our study, BCV may reduce exercise load by positively impacting inefficient respiration within patients.

BCV is one type of NPV approach; positive pressure ventilation does not have a similar effect on pulmonary circulation. Negative pressure ventilators using a whole body-sized box are still used in Europe, but this type utilizes continuous external negative pressure regardless of inspiratory and expiratory phases of the respiratory cycle. In contrast, BCV used in our study affects only the thorax and supports both inspiration and expiration individually. Thus, its effects are different from those exerted by continuous NPV. We believe that the effects displayed in this study are unique to BCV.

NT-proBNP plays a pivotal role in predicting PH—a reduction of >15 % per year is associated with improved mortality [26, 27]. As NT-proBNP increases stress on the ventricular wall, a decrease in this protein confers afterload reduction within the right ventricle and/or adaptation of ventricles to stress [28]. As the period of examination was limited in the present study, adaptation was unlikely; thus, afterload reduction represents the most probable explanation.

Other limitations of the current study include the small sample size, especially for the restriction group. Furthermore, this study was not designed as a control trial because of various limitations in controlling patient conditions. Ethical problems would have arisen if the control group had right heart catheterization performed twice during the study period without therapeutic intervention with BCV. Finally, as catheterization procedures within each facility could only be scheduled on certain days of the week, BCV sessions frequently had to be rescheduled. In general, BCV sessions were performed on weekdays because of insufficient availability of human resources at weekends. As implementation of BCV was negatively affected by weekends, holidays and unexpected human resource problems, the number and timing of BCV sessions consequently varied.

It is possible that resting in hospital without any workload is associated with the observed reduction of patient blood pressures. However, it is unlikely that bed rest for approximately 2 weeks solely underlies the significant decreases observed in mPAP, RAP, PAOP, CI, mean BP and NT-proBNP in patients presenting with chronic respiratory disease for decades. Furthermore, none of the patients had worked for several years, remaining at home most days because of respiratory failure.

## Conclusion

A series of BCV sessions significantly decreased mPAP in this patient cohort. As improvement in oxygenation by BCV was transient, other factors—especially decreased cardiac overload—likely play a role. BCV may provide a promising approach for treating PH caused by chronic lung disease. However, additional studies are needed to define duration, frequency and long-term effects of BCV in a larger sample size. Further consideration of the influence of BCV on respiration and circulation is required, as well as determination of mechanisms underlying potential patient benefits.

## Electronic supplementary material

Below is the link to the electronic supplementary material.

Supplementary material 1 (PDF 66 kb)

Supplementary material 2 (MPG 1210 kb)

Supplementary material 3 (MPG 2610 kb)
