# Supplementary material for: Effects of extrathoracic mechanical ventilation on pulmonary hypertension secondary to lung disease
Source: J Anesth. 2016 Apr 18;30:663–70. doi: 10.1007/s00540-016-2172-7 (PMC4956720; doi:10.1007/s00540-016-2172-7)
Supplement: Supplementary file 1 — Supplementary material 1 (PDF 66 kb) [file 540_2016_2172_MOESM1_ESM.pdf]

Effects of extrathoracic mechanical ventilation on pulmonary hypertension from lung disease

Journal of Anesthesia

Yoko Sato, Noriyuki Saeki, Takuma Asakura, Kazutetsu Aoshiba, Toru Kotani

Corresponding Author: Yoko Sato, MD

Department of Anesthesiology and Intensive Care Medicine, Tokyo Women's Medical University, E-mail: yok.s@nifty.com

Online figure legend

Video 1a The CT appearance around thoracic diaphragm of spontaneously by healthy volunteer

Video 1b The CT appearance around thoracic diaphragm control mode of BCV by healthy volunteer
